# Human Liver Stem Cells Suppress T-Cell Proliferation, NK Activity, and Dendritic Cell Differentiation

**DOI:** 10.1155/2016/8468549

**Published:** 2016-04-04

**Authors:** Stefania Bruno, Cristina Grange, Marta Tapparo, Chiara Pasquino, Renato Romagnoli, Ennia Dametto, Antonio Amoroso, Ciro Tetta, Giovanni Camussi

**Affiliations:** ^1^Department of Molecular Biotechnology and Health Science, University of Torino, 10126 Torino, Italy; ^2^Department of Medical Sciences, University of Torino, 10126 Torino, Italy; ^3^Liver Transplantation Center, University of Torino, Torino, Italy; ^4^Regional Transplantation Center, Piedmont, Molinette Hospital, Torino, Italy; ^5^Unicyte AG, Oberdorf, Switzerland

## Abstract

Human liver stem cells (HLSCs) are a mesenchymal stromal cell-like population resident in the adult liver. Preclinical studies indicate that HLSCs could be a good candidate for cell therapy. The aim of the present study was to evaluate the immunogenicity and the immunomodulatory properties of HLSCs on T-lymphocytes, natural killer cells (NKs), and dendritic cells (DCs) in allogeneic experimental settings. We found that HLSCs inhibited T-cell proliferation by a mechanism independent of cell contact and dependent on the release of prostaglandin E2 (PGE_2_) and on indoleamine 2,3-dioxygenase activity. When compared with mesenchymal stromal cells (MSCs), HLSCs were more efficient in inhibiting T-cell proliferation. At variance with MSCs, HLSCs did not elicit NK degranulation. Moreover, HLSCs inhibited NK degranulation against K562, a NK-sensitive target, by a mechanism dependent on HLA-G release. When tested on DC generation from monocytes, HLSCs were found to impair DC differentiation and DCs ability to induce T-cell proliferation through PGE_2_. This study shows that HLSCs have immunomodulatory properties similar to MSCs, but, at variance with MSCs, they do not elicit a NK response.

## 1. Introduction

Human liver stem cells (HLSCs) have been identified and characterized as a mesenchymal stromal cell-like population derived from adult human liver [1]. This population possesses many surface markers in common with bone marrow-derived mesenchymal stromal cells (MSCs) such as CD29, CD73, CD44, CD105, and CD146; in addition, they express specific hepatic cell markers (albumin, cytokeratin-8, and cytokeratin-18) and embryonic markers (Nanog, Oct3/4, Sox2, Musashi, SSEA4, and Pax2) [1, 2]. HLSCs are able to differentiate into osteocytes, endothelial cells, hepatocytes, and beta-like cells [1–3]. When injected in different experimental models of liver failure, HLSCs engraft in injured liver and improve liver function and morphology [1, 2]. In particular, HLSCs have been described to protect SCID mice with fulminant liver failure from death by direct differentiation into hepatocytes and by secreting soluble factors that limit the injury [2]. Moreover, HLSCs are able to improve recovery also of different organs, such as kidneys [4]. Being potential candidates for clinical application, it is important to investigate the immunogenicity of HLSCs and their interaction with the immune system. In fact, a common complication in allogeneic cell therapies is the immune rejection. However, therapy with allogeneic MSCs of different origin is facilitated by their low immunogenicity and immunomodulatory properties [5, 6].

The aim of the present study was to evaluate whether HLSCs could modulate the behavior of immune cells, in comparison with MSCs. In particular, we investigated the effects of HLSCs on T-lymphocytes, natural killer cells (NKs), and dendritic cells (DCs) in allogeneic experimental settings.

## 2. Materials and Methods

### 2.1. Culture of HLSCs and MSCs

HLSCs were isolated from human cryopreserved normal adult hepatocytes purchased from Lonza (Basel, Switzerland) and were cultured as described [1–4]. Briefly, hepatocytes were initially cultured for 2 weeks in hepatozyme-SFM medium at a density of 1.0–1.5 × 10^5^ viable cells per cm^2^. After 2 weeks, most of the hepatocytes died, and then medium was substituted by *α*-MEM/EBM-1 (3 : 1) (Lonza) media supplemented with l-glutamine (5 mM), HEPES (12 mM, pH 7.4), penicillin (50 IU/mL), streptomycin (50 *μ*g/mL) (all from Sigma, St. Louis, MO, USA), and fetal calf serum (FCS) (10%) (Invitrogen). When HLSC colonies were evident, cells were expanded and characterized. HLSCs expressed some MSC markers (CD29, CD44, CD73, CD90, and CD105), specific hepatic markers (albumin, *α*-fetoprotein, cytokeratin-8, and cytokeratin-18), and some embryonic stem cell markers (Oct3/4, Nanog, Pax2, Musashi, SSEA4, and Sox2) as previously described [1–3]. By cytofluorimetric analysis (FACS), conducted with FACSCalibur cytometer (Becton Dickinson Bioscience, San José, CA), the expression of costimulatory molecules and histocompatibility antigens, CD40, CD80, CD86, HLA class I, and HLA-DR (all from Becton Dickinson), was evaluated (dilution 1 : 20).

HLSCs obtained from each preparation showed the same phenotype and they were used within the 10th passages.

The expression of human leukocyte antigen G (HLA-G, dilution 1 : 20) on HLSCs was evaluated by FACS analyses using the specific MEMG/9 antibody (native form for human HLA-G1, EXBIO, Praha, Czech Republic). Extracellular staining and intracellular staining were used to study membrane-bound and intracytoplasmic HLA-G protein.

Bone marrow MSCs were purchased from Lonza, cultured, and characterized as previously described [2].

### 2.2. Peripheral Blood Mononuclear Cells Isolation

Peripheral blood mononuclear cells (PBMCs) were obtained from healthy donors (*n* = 3 males; *n* = 7 female and age 25–40 years old). All subjects gave informed consent and the study was approved by the local Ethical Review Board. PBMCs were isolated by centrifugation over Histopaque-1077 (Sigma).

### 2.3. T-Cell Purification and Culture

CD3-positive cells were sorted from PBMCs using immunomagnetic beads (Miltenyi Biotec, Cologne, Germany) and used in coculture experiments. PBMCs were characterized to typify HLA Class I and HLA Class II expression (Supplementary Table 1 in Supplementary Material available online at http://dx.doi.org/10.1155/2016/8468549). To this purpose DNA was extracted and Luminex technology was used (One Lambda, Thermo Fisher). Luminex technology uses sequence-specific oligonucleotide probes bound to color-coded microbeads in order to identify HLA alleles encoded by the DNA sample. As shown in Supplementary Table 1, different donors had different HLA class I and HLA class II phenotype and were mismatched with HLSCs.

The purity of CD3^+^ was 98 ± 2% assessed by FACS analysis (not shown). CD3^+^ cells were directly used for coculture experiments or activated with 10 ng/mL of phorbol myristate acetate (PMA, Sigma) or with a mix of CD3 (1 *μ*g/mL, Miltenyi Biotec) and CD28 (2 *μ*g/mL, Miltenyi Biotec) antibodies. Cocultures were established in direct contact or by using transwells (1 *μ*m pore, Falcon, Becton Dickinson) in 6- or 24-well plates (Corning Incorporated, NY, USA). HLSCs and MSCs (1 × 10^5^/wells) were seeded into the lower compartment of the culture wells. Different amounts of lymphocytes were loaded in the upper inserts to obtain ratios of lymphocytes/HLSCs or MSCs of 1 : 1, 2 : 1, 5 : 1, and 10 : 1. In selected experiments, in noncontact conditions, specific chemical antagonists of indoleamine 2,3-dioxygenase (IDO) and of prostaglandin E2 (PGE_2_) such as 1-methyl-L-tryptophan (an inhibitor of IDO, Sigma, 0.5 mM), indomethacin (Cox-1 and Cox-2 inhibitor, 10 *μ*M, ICN Chemicals, Irvine, CA), and NS-398 (a specific Cox-2 inhibitor, 1 *μ*M, Cayman Chemicals, Ann Arbor, MI) were added to coculture (ratio lymphocytes/HLSCs 5 : 1). All the experiments were conducted in duplicate. The T-cell proliferation analysis was performed, after 3 days of incubation, with the Muse Count & Viability Kit (Millipore, Billerica, MA), following the manufacturer's instruction, and subsequently analysed by the Muse*™* Cell Analyzer (Merck Millipore, Massachusetts, USA). In particular, the Muse Count & Viability Reagent contains fluorescent DNA-binding dyes, which have differential permeability to viable and nonviable cells, and provides absolute cell count and viability data on cell suspensions. Both viable and nonviable cells are differentially stained based on their permeability to the DNA-binding dyes in the reagent. Data generated using the Muse Cell Analyzer with the Muse software provide the viable cell count (cells/mL); the total cell count (cells/mL); and the percentage of viability of sample.

The amount of PGE_2_ produced by HLSCs, in basal condition and after cocultivation, was evaluated by ELISA (R&D System, Minneapolis, MN).

### 2.4. NK Purification, Culture, and Degranulation Assay

Nonactivated NKs were obtained using the NK Cell Isolation Kit (Miltenyi Biotec) that is an indirect magnetic labelling system for the isolation of untouched NKs from human PBMC. The purity of NKs after the isolation was 95 ± 3% assessed by FACS analysis (not shown). By this system T-cells, B cells, stem cells, dendritic cells, monocytes, granulocytes, and erythroid cells were depleted using immunomagnetic beads.

After purification, NKs were maintained for 24 hours in the presence of 15 ng/mL of interleukin- (IL-) 15 (Sigma). After this incubation, NKs were cocultured in the presence of or MSCs or HLSCs (ratio NK/MSC or HLSC 5 : 1) by using direct coculture or in the transwell system. In selected experiments, to test the functional role of HLA-G, the specific neutralizing antibody 87-G (10 *μ*g/mL, EXBIO) was added in coculture experiments. After 4 days, NKs were harvested and tested in the degranulation assay. The degranulation assay is a highly sensitive method and its results are strictly correlated with NK cytotoxicity [7]. The surface expression of CD107a (Becton Dickinson) was analysed after 4 hours following activation of purified NKs with target cells (HLSCs, MSCs, and K562, ATCC LGC Standards, Sesto San Giovanni, Italy; original donor: female, 53 years old) at a 1 : 1 ratio in the presence of a FITC labelled CD107a and of GolgiStop*™* (Becton Dickinson). The GolgiStop contains monensin that prevents the acidification of endocytic vesicles avoiding the degradation of reinternalized CD107a proteins from the surface. Subsequently, NK cells were stained with APC labelled anti-CD56 (Becton Dickinson) and analysed by FACS to measure the percentage of CD107a positivity, as described [7, 8]. In addition, the viability of HLSCs and MSCs was tested using the Muse Annexin V & Dead Cell Kit (Millipore), which permits enumeration of cells in various stage of apoptosis (early and late). The viability of K562 cells was evaluated by staining with propidium iodide (PI, 5 *μ*g/mL, Sigma). In this case, the K562 cells were labelled with green fluorescent dye carboxyfluorescein diacetate succinimidyl ester (CFSE, Molecular Probes, Invitrogen), to discriminate K562 green cells from NKs cells by FACS analyses.

### 2.5. Human Monocyte Isolation and Differentiation in DCs

Monocytes were isolated by plastic adherence. PBMCs were plated at a concentration of 5 × 10^6^ cell/mL in RPMI supplemented with 10% FCS in 6-well flat-bottomed plates and left to adhere in a 5% CO_2_ incubator at 37°C overnight. Nonadherent cells were removed by 2 consecutive washes with RPMI plus 10% FCS [9].

The next day, the DC differentiation medium, composed by RPMI supplemented with 10% FCS, 20 ng/mL of IL-4 (Sigma), and 50 ng/mL of granulocyte-macrophage colony-stimulating factor (GM-CSF, Sigma), was added. The purity of the monocyte population was quantified through FACS analyses, by positive staining for CD14 (87 ± 7%, not shown). Monocytes were cocultured in the presence of 1 × 10^5^ HLSCs or MSCs seeded in transwells (1 *µ*m pore). After 2 days 1/3 of the medium was replaced and after 4 days 200 ng/mL of lipopolysaccharide (LPS, Sigma) was added to the culture. Complete differentiation was reached after 7 days. The same experimental setting was repeated in the presence of NS-398 (1 *μ*M). The amount of PGE_2_ produced after cocultivation was evaluated by ELISA.

The DC differentiation was assessed after 7 days of culture using FITC, PE, and APC conjugated antibodies (all from Becton Dickinson) for CD14, CD80, CD83, CD86, HLA-DR, CD1a, CD40, CD54, *α*4 integrin, and *α*5 integrin. Data were expressed as percentage of positive cells ± SD and as geometric mean of fluorescence intensity ± SD.

Completely differentiated DCs in the presence or the absence of HLSCs were treated with 50 *µ*g/mL of mitomycin C (Sigma) in order to block their proliferation and cultured in triplicate at a concentration of 2 × 10^4^ in 96-well flat-bottomed plate (Corning Incorporated) with 1 × 10^5^ allogeneic CD3^+^ lymphocytes. As controls, CD3^+^ cells were cultured alone or were treated with 10 ng/mL of PMA to induce T-cell proliferation. Proliferation rates were analysed after 2 days of coculture using 5-bromo-2′-deoxyuridine (BrdU) incorporation Kit (Roche, Basel, Switzerland). DNA synthesis was detected as incorporation of BrdU into cellular DNA. After 3 days of incubation, cells were then fixed with 0.5 M ethanol/HCl and incubated with nuclease to digest the DNA. BrdU incorporated into the DNA was detected using an anti-BrdU peroxidase-conjugated mAb and visualized with a soluble chromogenic substrate. Optical density was measured with an ELISA reader at 405 nm.

### 2.6. RNA Extraction and Real Time Analysis

RNA was extracted by Trizol (Life Technology, Carlsbad, CA) and 200 ng of total RNA was retrotranscribed using High Capacity cDNA Reverse Transcription Kit (Applied Biosystems, Foster City, CA). RT-PCR mix containing 1x SYBR GREEN PCR Master Mix (Applied Biosystems), 100 nM of each primer, listed in Table 1, and 5 ng of cDNA were assembled using a 96-well StepOne Real Time System (Applied Biosystems). Negative cDNA controls (no cDNA) were cycled in parallel with each run.

Data are expressed as relative quantification using the ΔΔCt method. Normalization was made using actin as housekeeping gene (Table 1).

### 2.7. Western Blot and Immunoprecipitation for HLA-G

HLSCs were lysed for 15 minutes in a cold buffer containing 1% Igepal, 5 mM EDTA, 150 mM NaCl, 50 mM Tris-HCl (pH 8,0), 1 mM phenylmethylsulfonyl fluoride (PMSF), and a cocktail of protease and phosphatase inhibitors (all from Sigma). Western blots were performed on 10% SDS-PAGE (Bio-Rad, Hercules, CA). Nitrocellulose membranes (Bio-Rad Laboratories) were probed with Abs specific for HLA-G (1 : 200) and actin (1 : 300) (both from Santa Cruz Biotechnology) and with anti-mouse or anti-rabbit horseradish peroxidase- (HRP-) conjugated secondary Abs (1 : 3000) (Pierce, Waltham, MA) and developed using ECL plus detection reagents (GE Healthcare, Piscataway, NJ). Densitometry analysis was performed using Quantity One image acquisition and analysis software (Bio-Rad Laboratories).

For immunoprecipitation analysis, the supernatants collected from HLSCs were subjected to isopropanol precipitation (1 : 4) overnight at −80°C and then lysed with Ripa buffer. Secondary antibodies were preincubated with protein A/G plus agarose (Santa Cruz Biotechnology, Dallas, Texas, USA) for 1 h at 4°C to exclude nonspecific binding of proteins. The tagged proteins were immunoprecipitated with appropriate antibodies for 1 h at 4°C and then with protein A/G plus agarose overnight in a rotor at 4°C. After centrifugation for 3 times in freshly prepared NP-40 buffer (50 mM Tris-HCl, 250 mM NaCl, 2 mM EDTA, 50 mM NaF, and 0,1 mM Na_3_VO_4_), the pellets were used for SDS-PAGE (4%–20% acrylamide gel, Bio-Rad).

### 2.8. Statistical Analyses

Statistical analysis was performed with SPSS (IBM SPSS Statistics, version 20.0.0). Continuous variables are presented as mean ± standard deviation or as median (min-max), according to their distribution. The difference between groups was analysed with Student's *t*-test (unpaired, 2-tailed), one-way ANOVA, or ANOVA with Dunnet's multiple comparison test and, when necessary, with Bonferroni correction or with the nonparametric Wilcoxon test. Significance level for all tests was set at *p* < 0.05.

## 3. Results

### 3.1. HLSCs Suppress T-Cell Proliferation* In Vitro*

HLSCs are similar to MSCs in that they constitutively express HLA class I antigens but do not express HLA-DR and CD40, CD80, and CD86 costimulatory molecules.

**Figure 1:**
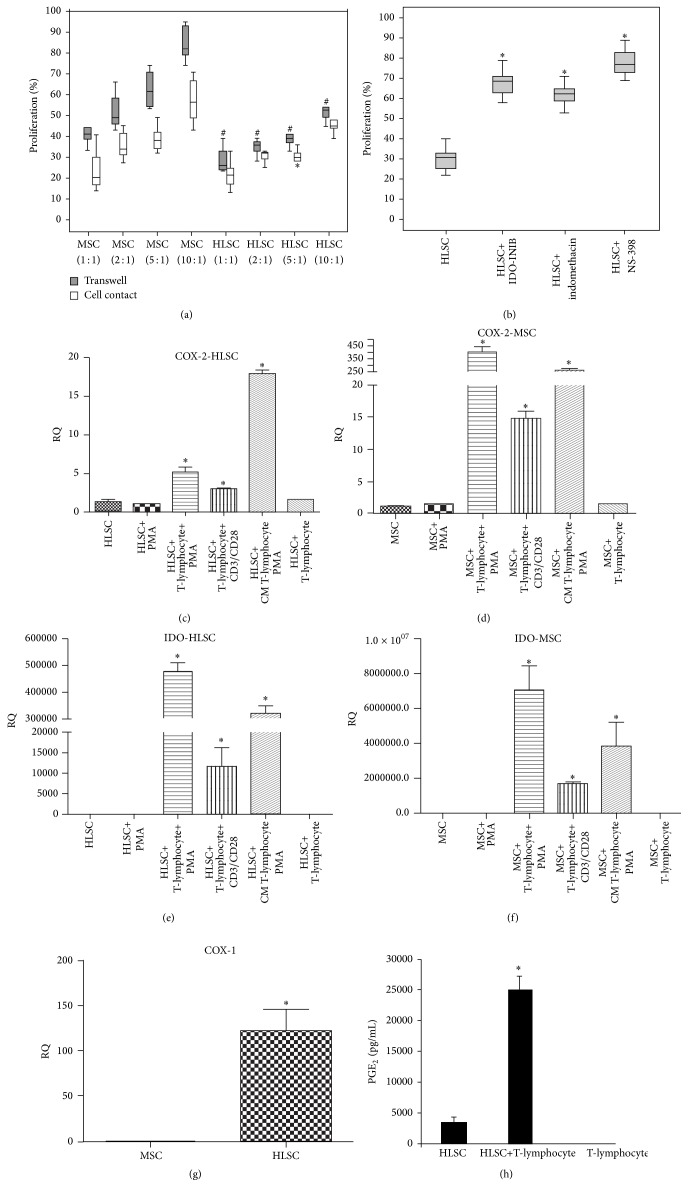
HLSCs suppress T-lymphocyte proliferation through IDO and PGE_2_ activity. (a) CD3^+^ lymphocytes were stimulated with PMA, in the presence or the absence of MSCs or HLSCs at different ratio (lymphocytes: HLSCs/MSCs 1 : 1, 2 : 1, 5 : 1, and 10 : 1) in direct contact or in the presence of transwells. The proliferation rate was evaluated after 3 days of coculture. Results are expressed as mean ± SD of 6 different experiments conducted in duplicate. 100% of proliferation corresponds to lymphocytes stimulated with PMA. Data were analysed by nonparametric Wilcoxon test: ^*∗*^ *p* < 0.05 lymphocytes stimulated with PMA cocultured in contact with HLSCs versus lymphocytes stimulated with PMA cocultured in contact with MSCs; ^#^ *p* < 0.05 lymphocytes stimulated with PMA cocultured with HLSCs, in the presence of transwells, versus lymphocytes stimulated with PMA cocultured with MSCs, in the presence of transwells. (b) IDO and PGE_2_ are involved in HLSC suppression of T-cell proliferation. CD3^+^ cells were stimulated with PMA in the presence of HLSCs (5 : 1 ratio) with or without inhibitors of IDO (1-methyl-L-tryptophan) and Cox-1 and/or Cox-2 (indomethacin, NS-398) in the presence of transwells. The proliferation rate was evaluated after 3 days. Results are expressed as mean ± SD of 6 different experiments in duplicate. 100% of proliferation corresponds to lymphocytes stimulated with PMA. Data were analysed by ANOVA with Bonferroni correction: ^*∗*^ *p* < 0.05 lymphocytes stimulated with PMA cocultured with HLSCs and different inhibitors versus lymphocytes stimulated with PMA cocultured with HLSCs. (c) and (d) mRNA expression levels of Cox-2 were evaluated in HLSCs (c) and MSCs (d) unstimulated, stimulated with PMA, after 3 days of coculture with CD3^+^ lymphocytes stimulated with PMA or with CD3/CD28 antibodies, stimulated with CM from T-cells stimulated with PMA, and cultured with unstimulated lymphocytes. (e) and (f) mRNA expression levels of IDO were evaluated in HLSCs (e) and MSCs (f) unstimulated, stimulated with PMA, after 3 days of coculture with CD3^+^ lymphocytes stimulated with PMA or with CD3/CD28 antibodies, stimulated with CM from T-cells stimulated with PMA, and cultured with unstimulated lymphocytes. Cox-2 and IDO expression levels increased after coculture with activated lymphocytes and after stimulation with CM. Data are expressed as relative quantification using ΔΔCt method. Normalization was made using actin as housekeeping gene. Data were analysed by ANOVA with Bonferroni correction, ^*∗*^ *p* < 0.05 gene expression of MSCs or HLSCs after coculture with CD3^+^ cells stimulated with PMA or CD3/CD28 antibodies or after culture with CM versus MSCs or HLSCs alone. (g) mRNA expression levels of COX-1 were evaluated in MSCs and HLSCs. Data are expressed as relative quantification using ΔΔCt method. Normalization was made using actin as housekeeping gene. Data were analysed by Student's *t*-test (unpaired, 2-tailed); ^*∗*^ *p* < 0.05 COX-1 expression of HLSCs versus MSCs. (h) Cell supernatants from cocultures were harvested to detect PGE_2_ production by ELISA. A significant increased production of PGE_2_ (*p* < 0.05) was observed after 3 days of coculture of CD3^+^ cells stimulated with PMA and cocultured with HLSCs.

To determine the capacity of HLSCs to interfere with T-lymphocyte proliferation, HLSCs were cocultured with allogeneic CD3^+^ cells activated or not with PMA. No proliferation was noted when purified T-lymphocytes were cocultured in the presence of HLSCs or MSCs, at different ratios (Supplementary Figure 1). Indeed, after 3 days of coculture, HLSCs inhibited PMA-induced CD3^+^ cell proliferation in a dose-dependent manner. In cell contact conditions, at a 5 : 1 lymphocyte : stem cell ratio, and in the presence of transwells, the inhibitory effect of HLSCs was significantly greater than that of MSCs (Figure 1(a) and Supplementary Figure 1). Cell contact was not required for HLSC suppression of T-cell proliferation, as HLSCs separated by a transwell were almost as effective as those in direct contact with lymphocytes (Figure 1(a) and Supplementary Figure 1). To confirm the capacity of HLSCs to interfere with T-cell proliferation, we also stimulated transwell cultures with anti-CD3 and CD28 antibodies and observed an inhibition of T-cell proliferation in the presence of HLSCs at different cell ratios (Supplementary Figure 2).

The immunomodulatory effect of MSCs has been previously related to the production of two key soluble factors IDO and PGE_2_. To investigate the relevance of these factors in HLSC suppression of T-cell proliferation, chemical antagonists were added to lymphocyte-HLSC transwell cocultures. The addition of 1-methyl-L-tryptophan (IDO-inhibitor) or indomethacin (Cox-1 and Cox-2 inhibitor) or NS-398 (Cox-2 inhibitor) significantly restored T-cell proliferation (Figure 1(c)). In the absence of HLSCs, these inhibitors did not interfere with T-lymphocyte proliferation (not shown). These data indicated that cell contact was not required and that Cox and IDO played a relevant role in the HLSC-induced suppression of T-cell proliferation. We therefore examined the mRNA levels of Cox-1 and Cox-2 and IDO in HLSCs and MSCs in basal condition and after coculture, in the presence of transwells. HLSCs constitutively expressed Cox-1 (Threshold cycle, Ct: 21 ± 1.2) and IDO (Ct: 31 ± 5.10). Moreover, Cox-1 expression is significantly more expressed in HLSCs in comparison with MSCs (Figure 1(g)). In both HLSCs and MSCs, the expression of Cox-2 and IDO mRNAs significantly increased after coculture with lymphocytes activated with PMA or with CD3 and CD28 antibodies (Figures 1(c)–1(f)). Nonactivated lymphocytes did not induce upregulation of mRNA expression levels of Cox-2 and IDO. We also tested if PMA might influence the expression levels of Cox-2 and IDO in HLSCs and MSCs. As shown in Figures 1(c)–1(f), PMA did not significantly increase the expression levels of Cox-2 and IDO. Instead, the conditioned medium (CM) derived from T-cells stimulated with PMA induced a significant increase in both MSCs and HLSCs. PGE_2_ production also significantly increased after culture with lymphocytes (Figure 1(h)). Stimulated T-lymphocytes in the absence of HLSCs did not produce PGE_2_ (Figure 1(h)).

### 3.2. HLSCs Do Not Stimulate NK Degranulation

**Figure 2:**
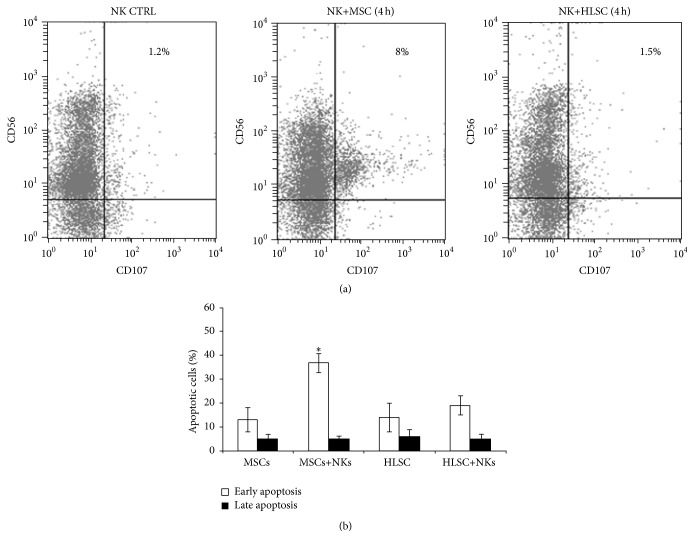
HLSCs used as target for NKs do not elicit NK degranulation. (a) Representative (*n* = 5) cytofluorimetric analysis of CD107a expression on NKs pretreated for 24 hours with IL-15 and incubated for 4 hours with MSCs or HLSCs, used as target cells in degranulation assay. CD107a expression was used to quantify cytotoxic granule exocytosis. After 4 hours of cocultivation with MSCs, a small percentage of NKs (about 8%) start to express CD107a; instead the NKs incubated with HLSCs did not degranulate and so did not express CD107a. (b) Early and late apoptosis of HLSCs and MSCs were evaluated after 4-hour incubation with NKs. Data are expressed as mean ± SD of percent variation of apoptotic cells. Results were obtained from 3 independent experiments performed in duplicate. Data were analysed by Student's *t*-test (unpaired, 2-tailed); ^*∗*^ *p* < 0.05 MSC cocultured with NKs versus control MSCs.

In order to compare the susceptibility of HLSCs to NK cell-mediated lysis, a degranulation assay was performed based on the detection of CD107a surface molecules on NK cell membranes, after NK-HLSC coculture. Allogeneic NKs were stimulated for 24 hours with IL-15 to increase their cytotoxic potential. Both HLSCs and MSCs were used as target cells.  Figure 2(a) shows that, after 4 hours of incubation, degranulation of NKs in response to HLSCs was absent (CD56^+^CD107^+^: 1 ± 0.5%). In contrast, MSCs induced NK degranulation (CD56^+^CD107^+^: 9.3 ± 3.2%), as previously described [8, 10, 11]. Moreover, the viability of HLSCs and MSCs was evaluated after 4 hours of incubation with IL-15 activated NKs. In these conditions, the percentage of early apoptotic HLSCs did not significantly increase, whereas the percentage of early apoptotic MSCs significantly increased (Figure 2(b)), confirming the data obtained by degranulation assay. No significant difference was observed in the percentage of late apoptotic cells between HLSCs and MSCs.

### 3.3. HLSCs Impair NK Function on Target Cells

**Figure 3:**
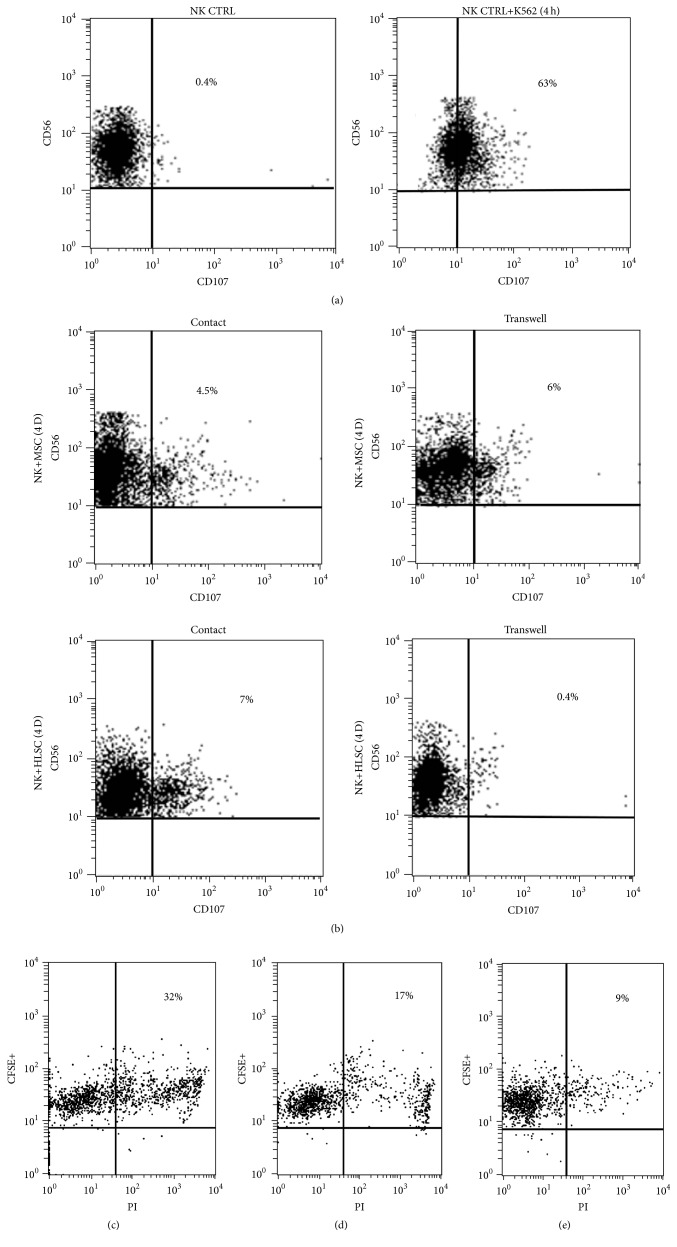
HLSCs inhibit NK activity. NKs, prestimulated for 24 hours with IL-15, were cocultured at the ratio of 5 : 1 with MSCs or HLSCs in direct contact or in transwells for 4 days. Subsequently, the NKs were challenged with a NK-susceptible target cell line (K562 cell line) and their degranulation was evaluated. (a) Representative (*n* = 3) cytofluorimetric analysis of CD107a expression on NKs (NK CTRL) and on NKs after 4 hours of incubation with K562 cells (NK CTRL+K562). CD107a was detectable on NK membrane after incubation with K562 cells. (b) Representative (*n* = 5) cytofluorimetric analysis of CD107a expression on NKs in the presence of K562 and after 4 days of incubation with MSCs or HLSCs. NKs cocultured with MSCs and HLSCs reduced their capacity to degranulate in the presence of specific target cells. (c)–(e) The death of K562 cells labelled with CFSE, which were evaluated by PI staining, after incubation for 4 hours with NKs stimulated with IL-15 (c) and cocultured, in the presence of transwells, for 4 days with MSCs (d) or with HLSCs (e).

To study whether HLSCs modulated NK activity, coculture experiments were carried out in direct contact or by using transwell inserts. Prestimulated NKs were cocultured with HLSCs or MSCs and subsequently harvested to evaluate the degranulation capacity against K562, a susceptible target cell line. K562 cells induced degranulation of NKs prestimulated with IL-15 (CD56^+^CD107^+^: 65 ± 8% (Figure 3(a))). Preincubation of NKs with HLSCs, in contact condition or in the presence of transwell, was able to significantly decrease NK degranulation (CD56^+^CD107^+^: resp., 8.2 ± 1.5% and 2.5 ± 2%), in a manner comparable with the preincubation with MSCs (CD56^+^CD107^+^: resp., 5.3 ± 2.2% and 7 ± 3%) (Figure 3(b)). Moreover, after incubation with HLSCs, NKs decreased their capacity to kill K562 cells (Figures 3(c) and 3(e)) and confirmed the data obtained by the degranulation test. Also NKs incubated with MSCs decreased their capacity to kill K562 cells (Figures 3(c) and 3(d)), but to a lesser extent than HLSCs (Figure 3(e)).

**Figure 4:**
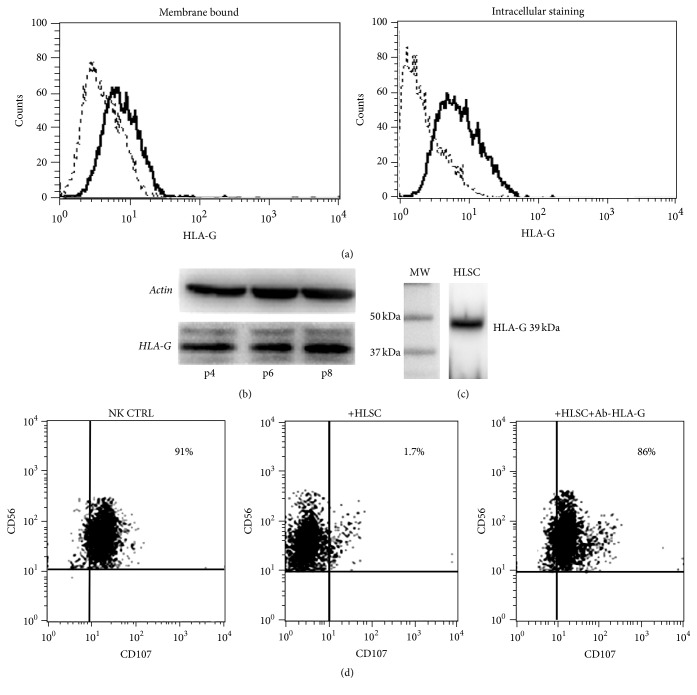
HLA-G expression mediates the HLSC activity on NKs. (a) Representative (*n* = 4) cytofluorimetric analysis of HLA-G expression on HLSC membrane and after intracytoplasmic staining, using the specific MEMG/9 antibody (native form for human HLA-G1). (b) Western Blot analyses confirmed the presence of HLA-G in HLSCs at different passages of culture (p4, p6, and p8). (c) Soluble HLA-G is present in HLSC supernatants, as indicated by Western Blot after immune-precipitation with specific antibody. HLA-G protein expression was detected through mAb 4H84, resulting in specific band corresponding to the expected molecular weight of 39 kDa. (d) Representative (*n* = 4) cytofluorimetric analysis of CD107a expression on NKs in degranulation assay, after 4 days of incubation with HLSCs in the presence or the absence of specific blocking antibody against HLA-G (87-G). HLSCs, via the secretion of HLA-G reduced NK degranulation, as a specific neutralizing anti-HLA-G antibody restored degranulation activity to levels similar to those following incubation of NKs with K562 cells alone (CD56^+^CD107^+^: 87 ± 5% in NK CTRL, 5.5 ± 4% in NKs cocultured with HLSCs, and 90 ± 5.3% in NKs cocultured with HLSCs in the presence of blocking antibody).

We investigated whether the expression of HLA-G molecule on HLSCs was involved in HLSC-induced inhibition of NKs. Intracellular HLA-G was detected by flow cytometry in HLSCs (69 ± 3.5%; Figure 4(a)) while only 10% of cells were positive for membrane-bound HLA-G staining. Western Blot analyses confirmed the presence of HLA-G in HLSCs at different passages of culture (Figure 4(b)). Soluble HLA-G was also present in HLSC supernatants (Figure 4(c)). Experiments with blocking antibody against HLA-G in transwells suggest that soluble HLA-G released by HLSCs is involved in the inhibitory effect on NK activity (Figure 4(d)).

### 3.4. HLSCs Suppress Monocyte-Derived DC Differentiation and Maturation

**Figure 5:**
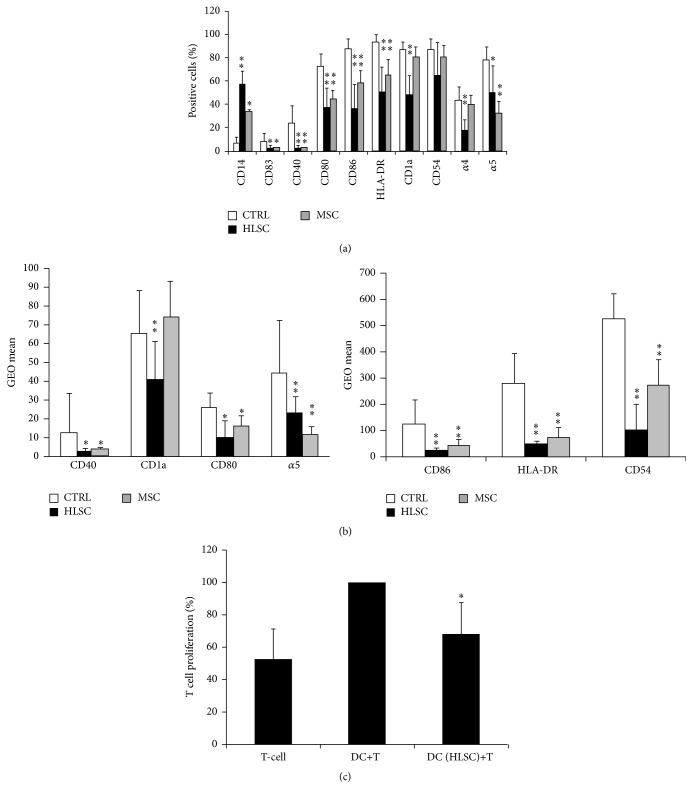
HLSCs suppress monocyte-derived DC differentiation and DC ability to stimulate T-cell proliferation. (a) Mean percentages ± SD of the expression of CD14, CD83, CD40, CD80, CD86, HLA-DR, CD1a, CD54, and *α*4 and *α*5 integrin of DCs differentiated in the presence of HLSCs or MSCs or in the absence of stem cells (CTRL) evaluated by cytofluorimetric analyses. Results were obtained from 8 independent experiments. ANOVA with Dunnet's multicomparison test was performed: ^*∗*^ *p* < 0.05 stem cells versus CTRL; ^*∗∗*^ *p* < 0.001 stem cells versus CTRL. (b) Mean fluorescence intensity expressed as GEO mean ± SD of CD40, CD1a, CD80, *α*5 integrin, CD86, HLA-DR, and CD54 of DCs differentiated in the presence of HLSCs or MSCs or in the absence of stem cells (CTRL). Results were obtained from 8 independent experiments. ANOVA with Dunnet's multicomparison test was performed: ^*∗*^ *p* < 0.05 stem cells versus CTRL; ^*∗∗*^ *p* < 0.001 stem cells versus CTRL. (c) DCs differentiated in the presence of HLSCs or in the absence were plated at cell concentration of 2 × 10^4^ with 1 × 10^5^ T CD3^+^ lymphocytes. Forty-eight hours later, T-cell proliferation was assessed. Data are expressed as mean ± SD of percent variation of T-cell proliferation in the presence or the absence of DCs differentiated in the presence of HLSCs with respect to T-cell proliferation in the presence of DCs alone (established as 100%). Results were obtained from 5 independent experiments. Data were analysed by Student's *t*-test (unpaired, 2-tailed); ^*∗*^ *p* < 0.05 DC (HLSC)+T versus DC+T.

Monocytes were cultured with GM-CSF and IL-4 in the presence or the absence of HLSCs or MSCs. After 4 days of culture, LPS was added to promote the complete differentiation of DCs. After 7 days of differentiation in these culture conditions, monocytes acquired the characteristic shape and morphology of DCs and their degrees of differentiation and maturation were assessed by FACS analyses. In DCs that differentiated in the absence of HLSCs and MSCs, the expression of CD14 (6 ± 4.8% of positive cells) surface marker was almost abrogated; CD80, CD86, and HLA-DR expression was high (72 ± 10%, 87 ± 8.6%, and 93 ± 6.9%, resp.) and the activation markers CD1a and CD40 were increased (87 ± 6.4%, 23 ± 15.0%, resp.) (Supplementary Figure 3 and Figure 5(a)). The presence of HLSCs cultured in transwells interfered the differentiation process of DCs, as shown by the persistence of CD14 (58 ± 10.5%) expression on monocyte-derived cells (Supplementary Figure 3 and Figure 5(a)) and inhibition of the differentiation and maturation markers such as CD80 (38 ± 16.1%), CD86 (37 ± 20.3%), HLA-DR (51 ± 21.3%), CD1a (45 ± 16.0%), and CD40 (2.8 ± 2.0%). Moreover, the expression of *α*4 integrin and *α*5 integrin adhesion molecules and CD54 decreased in the presence of HLSCs (Supplementary Figure 3 and Figure 5(a)). The mean fluorescence intensity (GEO mean) was also analysed for several surface markers. The intensity for CD80, CD86 and HLA-DR, CD54, and *α*5 integrin was significantly lower in DCs cultured with HLSCs compared with DCs differentiated in the absence of stem cells (Figure 5(b)). The effect of HLSCs on DC differentiation was comparable to that previously described for MSCs (Figures 5(a) and 5(b)) [12, 13]. Notably, the presence of HLSCs did not affect DCs viability (not shown) as already demonstrated for MSCs [12].

DCs promote antigen-specific T-cell activation. DCs stimulated with LPS were able to induce CD3^+^ lymphocyte proliferation. When DCs predifferentiated in the presence of HLSCs were used, a significant reduction of T-cell proliferation was observed (Figure 5(c)). These results confirm functional impairment of DC differentiation in the presence of HLSCs.

**Figure 6:**
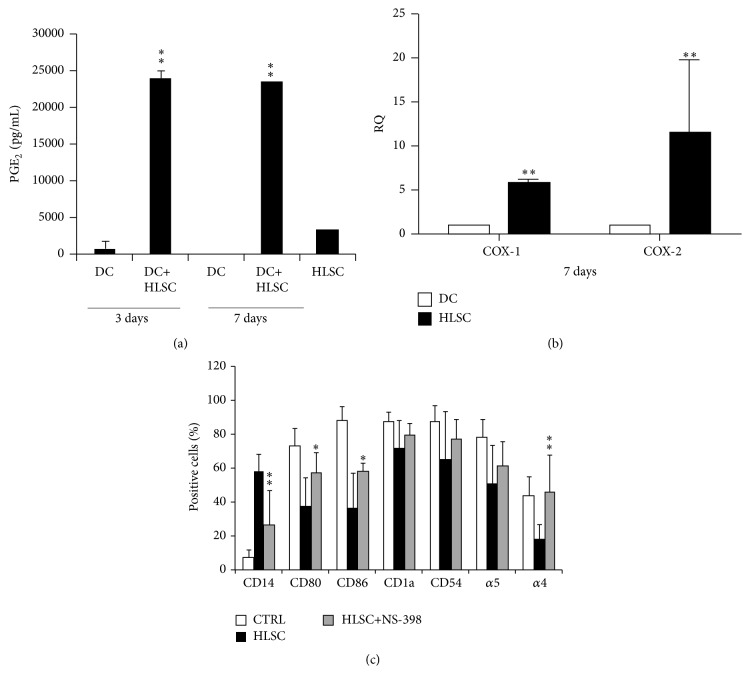
PGE_2_ mediates the HLSC activity on monocyte-derived DCs. (a) Cell supernatants were harvested to detect PGE_2_ production by ELISA after 3 and 7 days of coculture of DCs in the presence or the absence of HLSCs. ANOVA with Dunnet's multicomparison test was performed: ^*∗∗*^ *p* < 0.001 DC+HLSC versus HLSC. (b) mRNA expression levels of Cox-1 and Cox-2 were evaluated in HLSCs and in DCs cocultured for 7 days. Cox-1 and Cox-2 expression levels were higher in HLSCs compared with DCs. Data are expressed as relative quantification (RQ) using ΔΔCt method. Normalization was made using actin as housekeeping gene. ANOVA with Dunnet's multicomparison test was performed: ^*∗∗*^ *p* < 0.001 HLSC versus DC. (c) Mean of percentages ± SD of CD14, CD80, CD86, CD1a, CD54, and *α*4 and *α*5 integrin of positive DCs differentiated in the presence (HLSC) or in the absence (CTRL) of HLSCs or stimulated with NS-398. Results were obtained from 4 independent experiments. ANOVA with Dunnet's multicomparison test was performed: ^*∗*^ *p* < 0.05 HLSC versus HLSC+NS-398; ^*∗∗*^ *p* < 0.001 HLSC versus HLSC+NS-398.

The action of MSCs on DC differentiation has been ascribed to the production of many soluble factors, of which the most important seems to be PGE_2_ [14]. The presence of PGE_2_ was quantified by ELISA assay in supernatant of DCs differentiated in the presence or the absence of HLSCs (3 and 7 day of culture) and in HLSC supernatant (Figure 6(a)). As shown in Figure 6(a), HLSCs produced PGE_2_ constitutively; moreover, the amount of this mediator was significantly increased in coculture with DCs. DCs cultured alone did not produce PGE_2_ (Figure 6(a)). mRNA levels of Cox-1 and Cox-2 were evaluated in HLSCs and DCs when cocultured. mRNA levels of Cox-1 and Cox-2 were significantly higher in HLSCs compared with DCs, suggesting that PGE_2_ was mainly secreted by HLSCs (Figure 6(b)).

In addition, to test the biological role of PGE_2_, NS-398 was added to DC culture in the presence of HLSCs. PGE_2_ inhibition significantly reverted the negative effect of HLSCs on DC differentiation (Figure 6(c)). Taken together, these data suggest that PGE_2_ plays a central role in the inhibition of DC function.

## 4. Discussion

HLSCs are a population of liver stem-like cells easily obtainable from human adult liver [1–3]. They have a great proliferation potential and remain stable for over thirtieth culture passages, without acquiring chromosomal aberrations. Good manufacturing practice protocols to obtain and expand HLSCs have been established in our laboratory. Moreover, HLSCs have been approved by Italian Regulatory Agency for a phase I study, fulfilling all the release criteria for cellular therapies.

A critical issue in cell therapy is rejection, resulting from the immune-incompatibility between donor and recipient. In this paper, we show that HLSCs have immunomodulatory properties that may obviate the major obstacle for the application of these cells in cell therapy.

HLSCs did not express HLA class II and the costimulatory molecules CD40, CD80, and CD86. In addition, HLSCs inhibit (i) proliferation of mitogen-stimulated T-lymphocytes in a dose-dependent manner, (ii) degranulation capacity of NKs against a susceptible target cancer cell line, and (iii) DC differentiation and maturation.

The inhibitory effect of HLSCs on T-lymphocyte proliferation was dose-dependent (ratio lymphocytes to stem cells: 1 : 1, 2 : 1, 5 : 1, and 10 : 1) and was greater than that of MSCs. HLSC-dependent inhibition of T-cell proliferation was observed with a ratio up to 10 : 1. Furthermore, cell contact was not required for this effect, as HLSCs separated by a transwell were almost as effective as those in direct contact with lymphocytes. The mechanisms responsible for the suppression of T-lymphocyte proliferation also have been evaluated. HLSCs constitutively expressed Cox-1 and IDO; their mRNA levels increased after coculture with T-lymphocytes. Moreover, the addition of chemical antagonists of these enzymes significantly restored T-cell proliferation, indicating that Cox and IDO activities are involved in the antiproliferative effect of HLSCs. These observations are similar to that reported for MSCs [7, 15, 16]. It has been shown that PGE_2_ synthesis inhibition mitigated MSC-mediated suppression of T-lymphocytes [16, 17] and that IDO activity was involved in MSC-induced inhibition of T-cell proliferation [15].

Furthermore, it was found that HLSCs did not elicit a significant NK response. In contrast, MSCs were sensitive to NK as they induced high NK degranulation [8, 10, 11]. Therefore, HLSCs seem to be more protected than MSCs from allogeneic NK lysis. MSCs from other sources such as adipose tissue and embryonic stem cells have been also shown to be more resistant to NK lyses than bone marrow MSCs [8, 18, 19]. This may represent an advantage for their application in cellular therapies.

Another important aspect to be considered, related to NK/HLSC interaction, was whether the NKs cocultured with HLSCs modify their functionality over other susceptible target cells. The degranulation capacity of NKs on K562 cancer cell line was reduced after coculture with HLSCs also in the absence of contact. Similar inhibition of NK degranulation on target cells was observed for MSCs [11]. Previous studies demonstrated that the secretion of soluble HLA-G plays an important role in the biological effect of MSCs [20, 21]. HLA-G is a nonclassical HLA class I molecule with low polymorphism and a restricted tissue distribution (e.g., trophoblasts). HLA-G is known to confer to the fetus protection against the maternal immune system [22]. The addition of the specific blocking antibody against HLA-G to the NK/HLSC coculture prevented the inhibitory effect of HLSCs on NK degranulation. This observation, together with the presence of soluble HLA-G in HLSC supernatant, suggests a possible role of this molecule in NK modulation.

The effect of HLSCs on DC differentiation was also comparable to that previously described for MSCs [11–13, 23]. In the presence of HLSCs, the differentiation of CD14^+^ monocytes into DCs was impaired. In the appropriate culture condition and in the presence of HLSCs, monocytes retained the expression of CD14 and did not upregulate specific dendritic markers of maturation and activation. The addition of a specific PGE_2_ inhibitor reverted this effect suggesting that PGE_2_ secreted by HLSCs plays a central role in the inhibitory effect of HLSCs on DC differentiation. In addition, HLSCs also efficiently suppressed the ability of DCs to stimulate T-cell-activation and proliferation.

The immunomodulatory effect of HLSCs in physiological conditions is hard to demonstrate* in vivo* as this population has been described only in humans. In addition, the ratio of HLSCs/lymphocytes used* in vitro* possible does not represent ratio present in the human liver. However, if used therapeutically, the amount of HLSCs administered may account for an* in vivo* immunomodulatory effect as shown for MSCs which in patients with graft versus host disease are repeatedly injected at doses in the range of 1–1.5 × 10^6^ cells/Kg [24, 25].

## 5. Conclusions

The results of the present study indicate that HLSCs display optimal characteristics for cell therapy in an allogeneic setting. In fact, they modulate different aspects of the immune response including immunogenicity and T and NK responses. These results may be relevant for cell transplantation since these cells received the Orphan Drug designation from the European Medical Agency for ornithine transcarbamylase deficiency (EU/3/11/904), carbamoyl-phosphatase synthase deficiency (EU/3/12/971), and acute liver failure (EU/3/12/983).

## Supplementary Material

Supplementary Table 1. Phenotype of HLA I and II of HLSCs and of PBMCs derived from different healthy donors.
Supplementary Figure 1. HLSCs suppress T-lymphocyte proliferation induced by PMA.
Supplementary Figure 2. HLSCs suppress T-lymphocyte proliferation induced by CD3/CD28 antibodies.
Supplementary Figure 3. HLSCs suppress monocyte-derived DC differentiation.

## Figures and Tables

**Table 1:** List of primer sequences.

Gene name	Sequence
COX-1 forward	CTTGGGCCATGGGGTAGAC
COX-1 reverse	TCTACCGAGGGCGGGTACA
COX-2 forward	CTGGCAGGGTTGCTGGTG
COX-2 reverse	CTTCAGCATAAAGCGTTTGCG
IDO forward	GCCCTTCAAGTGTTTCACCAA
IDO reverse	GCCTTTCCAGCCAGACAAATAT
Actin forward	TGAAGATCAAGATCATTGCTCCTC
Actin reverse	CACATCTGCTGGAAGGTGGAC

## Abbreviations

HLSCs: Human liver stem cells
MSCs: Mesenchymal stromal cells
NKs: Natural killer cells
DCs: Dendritic cells
PBMCs: Peripheral blood mononuclear cells
PMA: Phorbol myristate acetate
IDO: Indoleamine 2,3-dioxygenase
PGE_2_: Prostaglandin E_2_.
