# Supplementary material for: Human Liver Stem Cells Suppress T-Cell Proliferation, NK Activity, and Dendritic Cell Differentiation
Source: Stem Cells Int. 2016 Apr 4;2016:8468549. doi: 10.1155/2016/8468549 (PMC4834412; doi:10.1155/2016/8468549)
Supplement: Supplementary file 1 — Supplementary Table 1. Phenotype of HLA I and II of HLSCs and of PBMCs derived from different healthy donors. Supplementary Figure 1. HLSCs suppress T-lymphocyte proliferation induced by PMA. Supplementary Figure 2. HLSCs suppress T-lymphocyte proliferation induced by CD3/CD28 antibodies. Supplementary Figure 3. HLSCs suppress monocyte-derived DC differentiation. [file 8468549.f1.pdf]

Supplementary Table 1. Phenotype of HLA I and II of HLSCs and of PBMCs derived from different healthy donors.

	CLASS I			CLASS II		
	**HLA-A**	**HLA-B**	**HLA-C**	**HLA-DRB1**	**HLA-DQA1**	**HLA-DQB1**
**HLSCs**	03, 32	44	04, 05	03 ,13	01, 05	02, 06
**DONOR 1**	03, 26	13, 55	03, 06	04, 07	02, 03	02, 03
**DONOR 2**	02, 68	07, 50	06, 07	07, 15	01, 02	02, 06
**DONOR 3**	02, 03	18, 35	04, 07	11	05	03
**DONOR 4**	02, 24	18, 51	02, 12	01, 11	01, 05	03, 05
**DONOR 5**	02, 03	07, 57	06, 07	03, 07	02, 05	02
**DONOR 6**	02, 33	14, 51	04, 08	01, 14	01	05

SUPPLEMENTARY FIGURES AND LEGENDS.

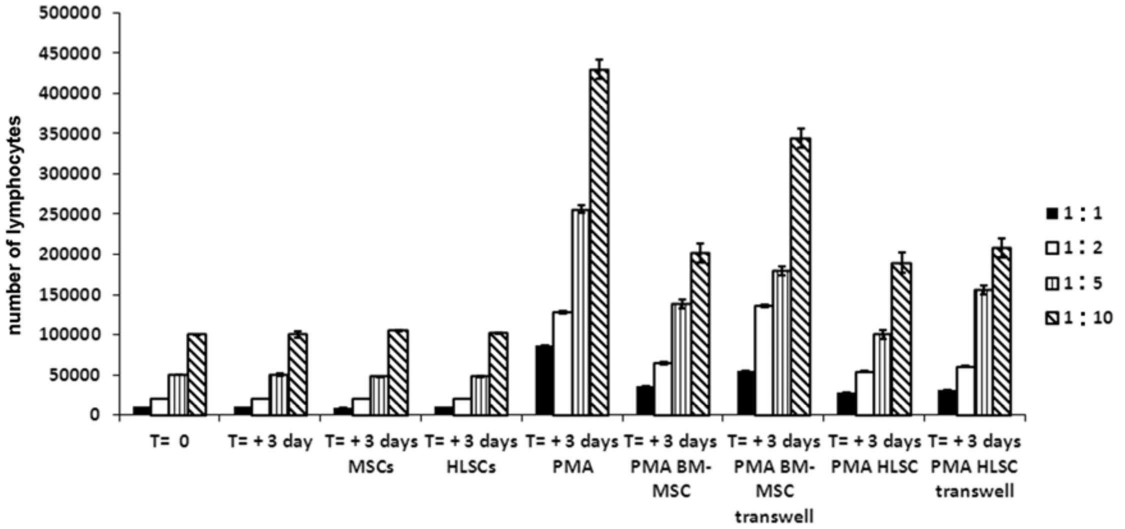

Supplementary Figure 1

Supplementary Figure 1. HLSCs suppress T-lymphocyte proliferation induced by PMA. $CD3^+$ lymphocytes were stimulated with PMA, in the presence or absence of MSCs or HLSCs at different ratio (lymphocytes: HLSCs/MSCs 1:1, 2:1, 5:1, 10:1) in direct contact or in the presence of transwells. The proliferation rate was evaluated after 3 days of co-culture. Results are expressed as mean ±SE of absolute number of T cells in one representative experiment conducted in duplicate. Six experiments with similar results have been conducted.

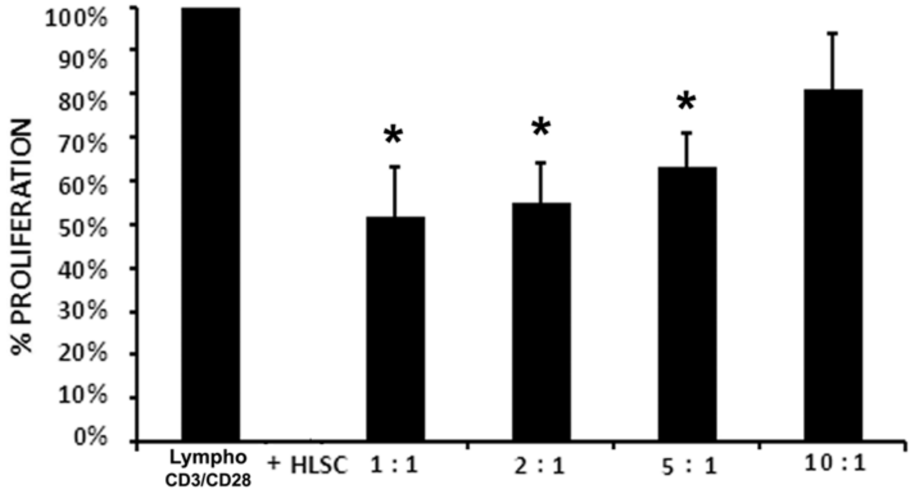

Supplementary Figure 2

Supplementary Figure 2 . HLSCs suppress T-lymphocyte proliferation induced by CD3/CD28 antibodies. $CD3^+$ lymphocytes were stimulated with anti-CD3/CD28 antibodies, in the presence or absence of HLSCs at different ratio (lymphocytes: HLSCs 1:1, 2:1, 5:1, 10:1) in the presence of transwells. The proliferation of T cells was evaluated after 3 days of co-culture. Results are expressed as mean ±SD of 3 different experiments conducted in duplicate. 100% of proliferation correspond to lymphocytes stimulated with CD3/CD28 antibodies. Data were analysed by ANOVA with Bonferroni correction; * $p < 0.05$ lymphocytes stimulated with CD3/CD28 co-cultured with HLSCs at different ratio vs lymphocytes stimulated with CD3/CD28 alone.

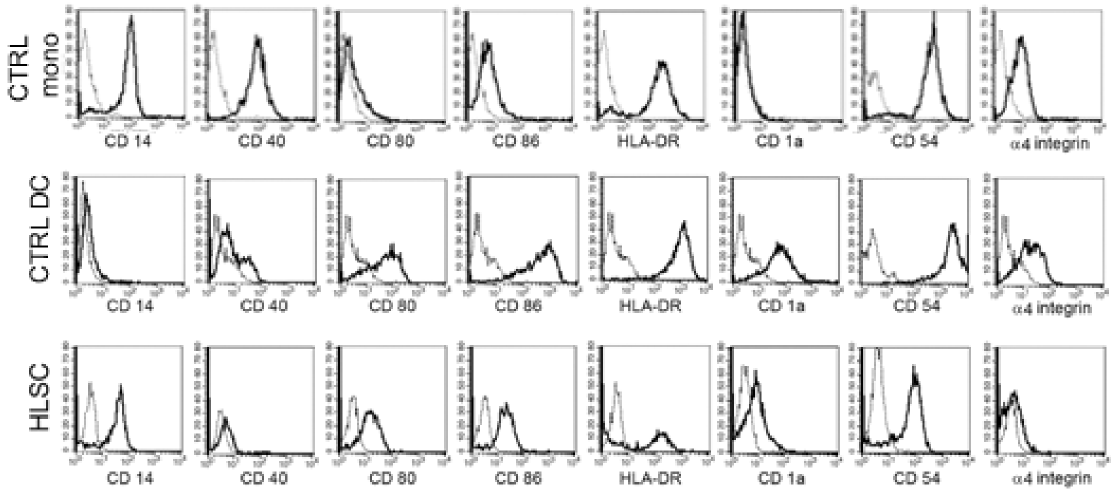

Supplementary Figure 3

Supplementary Figure 3. HLSCs suppress monocyte-derived DC differentiation. Representative cytofluorimetric analysis of monocyte (CTRL mono) and monocyte-derived DCs differentiated in presence or absence (CTRL DC) of HLSCs showing the expression of the following surface markers: CD14, CD40, CD80, CD86, HLA-DR, CD1a, CD54 and α4 integrin.
